# The regulatory role of affective inhibitory control in somatic symptoms among adolescents exposed to child maltreatment: a population-based study

**DOI:** 10.1007/s00787-022-01988-7

**Published:** 2022-04-20

**Authors:** Sjur Skjørshammer Sætren, Else-Marie Augusti, Mia Cathrine Myhre, Gertrud Sofie Hafstad

**Affiliations:** 1 Department for Child and Adolescent Research, Norwegian Centre for Violence and Traumatic Stress Studies, Oslo, Norway; 2 TIPS Centre for Clinical Research in Psychosis, Stavanger University Hospital, Jan Johnsens gate 12, 4011 Stavanger, Norway

**Keywords:** Emotion regulation, Inhibitory control, Adolescents, Child maltreatment, Somatic symptoms, Population-based study

## Abstract

Adolescents exposed to child maltreatment are at increased risk for various somatic symptoms, but which psychological factors that contribute to this relationship need to be further investigated. Emotion dysregulation is suggested to serve as a proximal link between child maltreatment and somatic complaints. The purpose of the present study was to investigate whether individual differences in affective inhibitory control, a central component in implicit emotion regulation, contribute to the risk of somatic symptoms in adolescents exposed to child maltreatment. Data were drawn from the UEVO study, a national population-based survey of adolescents between 12 and 16 years of age (*N* = 9240). For this study, we included participants who completed the emotional go/no-go task measuring affective inhibitory control (*N* = 7241; *M*_age_/SD = 14 years/.87; 52% girls, 47% boys), of which *N* = 3349 reported at least one incident of maltreatment exposure (57% girls, 41% boys). Exposure to psychological abuse and sexual abuse were associated with somatic symptoms. Affective inhibitory control was related to somatic symptoms, both in the total sample and in adolescents exposed to child maltreatment. The strength of relationships between exposure to psychological abuse and somatic symptoms, as well as sexual abuse and somatic symptoms, were moderated by individual differences in affective inhibitory control problems. Our study suggests that psychological abuse and sexual abuse increase the risk for somatic symptoms in adolescence. Affective inhibitory control, a central component in implicit emotion regulation, was related to somatic symptoms and moderated the relationships between psychological abuse and somatic symptoms, and sexual abuse and somatic symptoms. Revealing these associations in a population-based sample indicates that treatment targeting affective inhibitory control may be beneficial and should be explored further in clinical settings.

**Supplementary Information:**

The online version contains supplementary material available at 10.1007/s00787-022-01988-7.

## Introduction

Adolescents exposed to child maltreatment are at greater risk for developing somatic symptoms [1–4]. In many cases, symptoms remain medically unexplained, depriving individuals of targeted treatment and resulting in an increased risk for developing chronic symptom patterns that interfere with daily functioning [4, 5]. Chronic pain (abdominal, back, joint, head, and chest), fatigue and gastrointestinal problems are the most frequently reported somatic symptoms in children and adolescents [6]. The relationship between child maltreatment and somatic symptoms is complex, both with regard to how different features of abusive experiences are related to symptoms, but also in what way resilience or risk factors modulate this relationship. Different types of child maltreatment, such as physical, psychological or sexual abuse often co-occur, and some studies suggest that collectively these represent an equal risk for internalizing psychopathology, including somatic symptoms [7]. However, the majority of studies investigating child maltreatment and somatic symptoms in adolescents have focused on sexual abuse and physical abuse while fewer have taken other forms of abuse, such as psychological abuse or witnessing domestic violence into account [2, 8, 9]. Increasing evidence suggests that exposure to psychological and sexual abuse in particular, increases the risk for somatic symptoms when adjusting for co-occurrence of other forms of abuse [1].

Adolescent emotion regulation (ER) is found to be an important modulating factor on the association between child maltreatment and internalizing psychopathology [10–12]. Internalizing psychopathology and somatic symptoms are closely related in adolescents, and most pronounced in girls [6], also in samples with a history of child maltreatment [13]. A growing body of evidence suggests that emotion dysregulation represents a risk factor in the etiology of somatic symptoms [14, 15]. However, studies on ER in adolescents exposed to child maltreatment have predominantly focused on explicit ER strategies [16, 17]. It has been highlighted that future work on the role of implicit ER in the development of mental health problems in adolescents exposed to child maltreatment is needed [17], as implicit ER is associated with the ability to implement adaptive ER strategies [18, 19].

### Implicit emotion regulation, affective control and somatic symptoms

ER encompasses several psychophysiological, cognitive and behavioral mechanisms involved in the explicit or implicit modulation of emotional responses [20–22]. Explicit ER involves a range of conscious and effortful regulatory processes, such as the use of ER strategies in face of emotion eliciting situations [22]. Implicit ER (or automatic ER) refers to regulatory processes that are evoked automatically by a stimulus, often without any awareness [22]. Together, both explicit and implicit ERs are crucial for flexible adaptation to continuous shifting environments [23], especially in the face of adversity [12]. Emotion dysregulation occurs when emotional responses interfere with goal-directed behavior and potentially impedes daily functioning. Theoretical models [24, 25] as well as empirical studies [15] acknowledge that emotion dysregulation contributes to the development and progression of somatic symptoms. Previous research shows that the use of maladaptive ER strategies, such as rumination or catastrophizing, is related to increased somatic symptoms after traumatic experiences [26]. Use of adaptive ER strategies on the other hand has been found to reduce the impact of early life adversity on somatic symptoms [27]. In line with this, psychological treatments targeting explicit ER can be highly effective for patients with persistent somatic symptoms [28]. However, compared to the numerous studies on explicit emotion dysregulation and somatic symptoms [15], few have investigated how implicit ER relates to somatic symptoms in adolescents exposed to child maltreatment.

Affective inhibitory control, a fundamental component of cognitive control in emotion processing [18], is crucial for implicit ER [18, 29–31], and refers to the capacity to attend and respond to goal-relevant information while inhibiting attentional and behavioral responses to distracting goal-irrelevant emotional information [18]. Previous research document the importance of affective inhibitory control for adolescent mental health [18, 32]. Reduced affective inhibitory control increases vulnerability for internalizing symptoms during adolescence, especially in girls [33, 34]. On the other hand, better inhibitory control may buffer against internalizing symptoms in high-risk adolescent populations [29]. Inhibitory capacity over emotional responses has also been suggested as a link between psychosomatics and psychopathology [25]. Furthermore, a small but increasing number of studies also suggest that adult patients suffering from persistent somatic symptoms display difficulties with affective inhibitory control [15, 35, 36]. The current evidence suggest that affective inhibitory control may contribute to the risk for somatic symptoms in adolescents exposed to maltreatment.

Adolescents with a history of maltreatment show exaggerated attention to threatening over neutral stimuli [37], which is adaptive in the context of danger as it facilitate protective responses. However, for adolescents with reduced affective inhibitory control, this may develop into maladaptive patterns of attention in safe environments, making it difficult to disengage attention away from potential threatening information when no danger is present [38]. This in turn may increase the risk for persistent elevated internalizing symptoms, including somatic symptoms [30]. Together, these findings are in line with cognitive theories of persistent somatic symptoms, which emphasize vigilant attention to symptom cues [39]. Nonetheless, it remains to be investigated whether affective inhibitory control influences the risk for somatic symptoms in adolescents with a history of child maltreatment, during a developmental time at which these process still undergo development [32].

### The present study

The present study investigated the association between child maltreatment and somatic symptoms, and the moderating role of affective inhibitory control on these relationships. The purpose was to shed light on emotion regulatory processes through which child maltreatment may translate into pathology [10–12, 26, 40]. We first hypothesized that heightened exposure to physical abuse, psychological abuse, witnessing domestic violence and sexual abuse, will all be associated with higher levels of somatic symptoms. We expect that psychological and sexual abuse will be more strongly associated with somatic symptoms than other forms of abuse [1]. Second, we hypothesized that problems with affective inhibitory control are associated with more self-reported somatic symptoms, both in the total sample and for adolescents exposed to child maltreatment. Third, we hypothesized that the strength of relationships between child maltreatment exposures and somatic symptoms will vary depending on adolescents’ affective inhibitory control. The relationship will be stronger for adolescents with reduced affective inhibitory control capacity, compared to adolescents with better inhibitory capacity.

## Methods

### Participants

The data for this study were drawn from the Norwegian Youth Study on Child Maltreatment (The UEVO study), a large-scale and nationwide web-based survey of adolescents’ exposure to child maltreatment [*N* = 9240; 41]. The data were collected in 2019 using stratified sampling, resulting in a representative sample of adolescents aged 12–16. For this study, we used data from participants who completed the behavioral task measuring affective inhibitory control (*N* = 7241, 57% girls). The behavioral task was programmed for pc and not compatible with tablets. Therefore, schools using tablets in completing the questionnaire were excluded from the present study sample. The sample included for this study is comparable to the original sample on all parameters [41]. The majority of the adolescents were born in Norway, with 16.8% representing an ethnic minority. A subgroup was selected based on self-reported experience of at least one incident of psychological, physical or sexual abuse, or of witnessing domestic violence (*N* = 3349, *M*_age_/SD = 14 years/0.85). We selected a comparison group of adolescents without a history of abuse (*N* = 3666, *M*_age_/SD = 14 years/0.87; 48% girls).

### Measures

#### Child maltreatment

The instruments measuring child maltreatment were developed as part of the UEVO study [41]. For a more detailed description of the instrument, see Hafstad, Sætren [41]. Witnessing domestic violence was measured using six behavior-specific items (e.g., caregiver being pushed, hit or beaten by their partner), with high internal consistency (α = 0.86. For physical abuse, a scale consisting of six behavior-specific items (α = 0.78) was used to capture experiences of being tugged/held, pushed, beaten with a flat hand, beaten with a fist, kicked or being beaten up by one or more caregivers living with the child. The scale measuring psychological abuse consisted of eight behavior-specific items (α = 0.70) capturing experiences of being harassed, threats of being abandoned, threats of being beaten or injured, being locked out and threats of harming a family pet by one or more caregivers living with the child. For child sexual abuse, six behavior-specific items (α = 0.72) were used to capture experiences of an adult showing private parts of their body, touching private parts of the child’s body or of having intercourse with a relative or non-relative adult. All items were presented on a four-point scale (0 = never, 1 = once, 2 = sometimes, and 3 = often), and responses are summarized as a total index score of exposure. Mean scores were computed for the analyses. A dichotomous variable of exposed vs. not exposed was also calculated to compare adolescents with and without a history of child maltreatment exposure.

#### Somatic symptoms

A short version of the Children’s Somatic Symptoms Inventory [CSSI; 42, 43] was used to assess somatic symptoms. Items from the validation study with adequate statistical properties were selected in collaboration with the authors of the CSSI-24 [42]. The scale includes eight items covering stomach pain, headache, back pain, arm or leg pain, faintness or dizziness, rapid heartbeat, nausea and fatigue. All items are presented on a four-point scale ranging from 0 (not bothered) to 3 (extremely bothered). A mean score was computed for participants’ responses using the half-rule (observations required for at least 50% of items). The Cronbach’s alpha for the scale was 0.83 for the total sample, indicating high internal consistency.

#### Affective inhibitory control

**Fig. 1:**
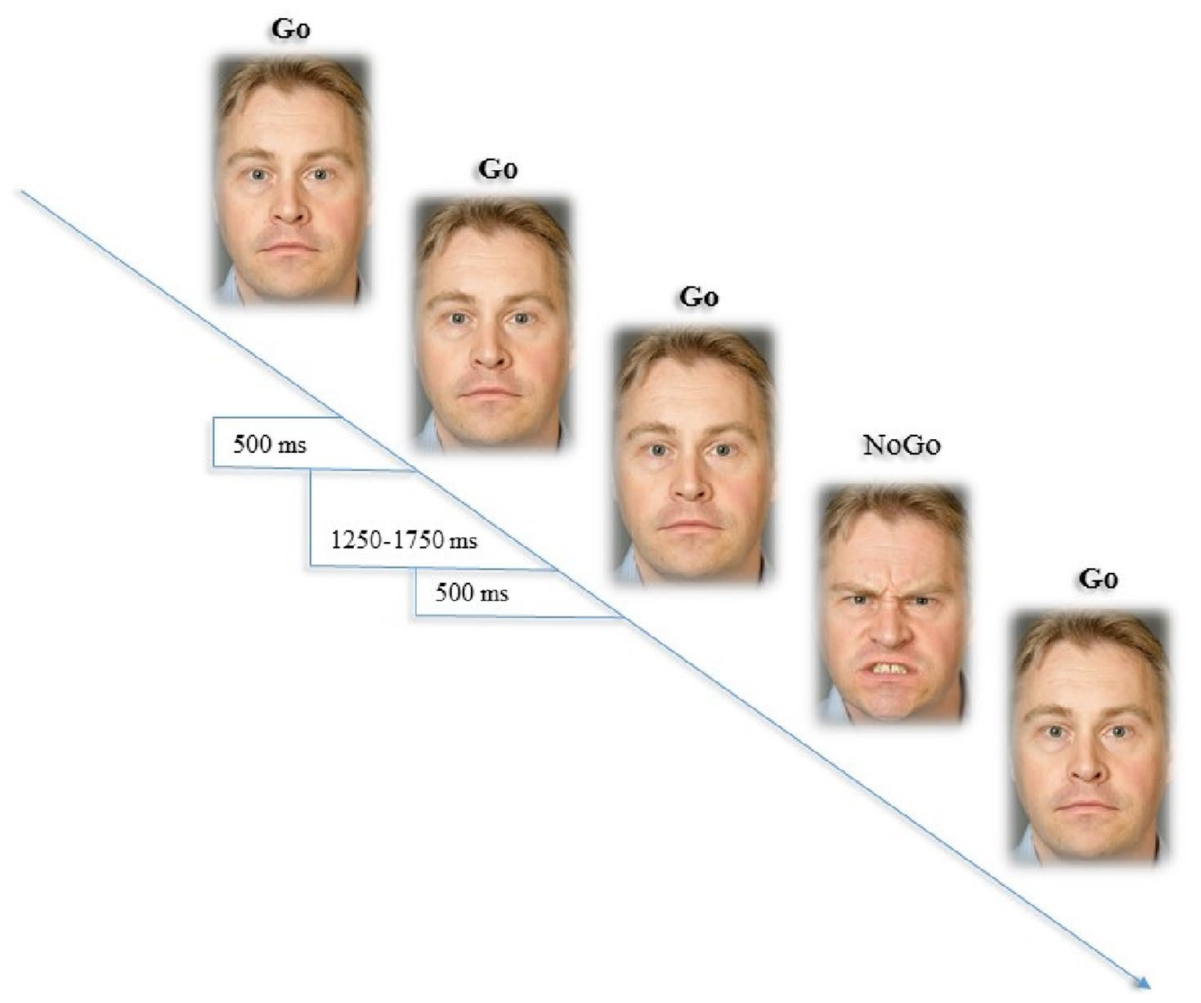
Visual illustration of stimulus presentation in the emotional go/no-go task

The emotional go/no-go task (Hare et al. 2008) was included in the survey as a behavioral task that measures affective inhibitory control. This self-administered, online-based task was programmed in Java and responses were collected by Conexus software (https://conexus.net/). All participants completed two blocks of the emotional go/no-go task using neutral and angry facial expressions as target and non-target stimuli (neutral/angry and angry/neutral). Each block consisted of 32 trials (22 go trails and 10 no-go trails). The order of target and non-target stimuli was pseudo-randomized and participants were told that they would be presented with different faces. Participants were instructed to respond as quickly as possible by pressing their keyboard’s spacebar on go trials while withholding (i.e., inhibiting) their responses on no-go trials. The facial expressions were displayed in the center of the screen, and a center point (+) was shown prior to the presentation of each stimuli. A disproportionately higher number of go trials (70/30 ratio) were presented to establish proponent and automatic responses to go trials. Each stimuli was displayed for 500 ms with an inter-stimulus interval varying randomly between 1250 and 1750 ms (see Fig. 1 for visual illustration).

For analyses using affective inhibitory control indices, false alarms (FA; i.e., commission errors) were calculated for both conditions: FA angry, when angry facial expressions were presented as distractive stimuli; and FA neutral, when neutral facial expressions were presented as distractive stimuli. FA angry has previously been used as an index of emotion dysregulation [44, 45].

### Statistical analyses

All statistical analyses were conducted using IBM SPSS Statistics for Windows, version 27.0. Descriptive statistics were calculated for age, gender, child maltreatment exposure and affective inhibitory control indices. Differences between maltreated and non-maltreated adolescents were explored by running *t* tests. Simple relationships between child maltreatment exposure, affective inhibitory control and somatic symptoms variables were investigated by Pearson correlation analysis.

To investigate our hypotheses, we conducted a series of multiple linear regression analyses, adjusting for age and gender. First, we ran a regression model investigating relationships between specific forms of child maltreatment exposure and somatic symptoms. Second, we examined whether affective inhibitory control and somatic symptoms were related, in the total sample and maltreatment sample. Third, to investigate whether the strength of the relationships between exposure to specific forms of child maltreatment and somatic symptoms interacted with individual differences on affective inhibitory control, we conducted moderation analyses using the PROCESS macro-version 3.5 for SPSS [46]. We ran moderation analyses when significant relationships between types of child maltreatment exposures and somatic symptoms were revealed. We conducted separate regression models for both task conditions (i.e., FA angry and FA neutral). Significant interaction effects were followed up by simple slope analyses investigating the conditional effects of FA angry and FA neutral on the relationship between child maltreatment exposure and somatic symptoms at 1 standard deviation (SD) below the mean, at the mean and 1 SD above the mean of FA angry/FA neutral. To adjust for non-normality in child maltreatment exposure and somatic symptoms we ran bootstrap tests with 1000 replications for all analyses [47].

### Ethics

The Regional Committee for Medical and Health Research Ethics in Norway (Case # 2018/522) approved this study. In line with a regulation in the *Act on Medical and Health Research* in Norway, participating adolescents were allowed to give their consent independent of their parents [48]. The study had a comprehensive post-survey follow-up procedure to assure that adolescents who perceived the survey to be stressful, or otherwise required attention after completing the survey, were taken care of.

## Results

Descriptive statistics for the 7241 participants are presented in Table 1, including separate results for both the maltreatment and comparison group. As presented in Table 1, we did not observe any meaningful differences (Cohen’s *d* < 0.10) between the maltreated group and the comparison group on the emotional go/no-go task [FA neutral: *t* (7013) = -3.889, *p* < 0.001]; FA angry *t* (7013) = − 3.518, *p* < 0.001). A significant difference with large effect size was observed for CSSI, where the maltreated group reported considerably more somatic symptoms than the non-maltreated group [*t* (6980) = − 31.307, *p* < 0.001].

**Table 1:** Descriptive statistics

Variable (range)	*N*	Total *N* = 7241	Maltreatment *N* = 3349	Comparison *N* = 3666	Cohen’s *d*
		Mean (SD)	Mean (SD)	Mean (SD)	
1. Age (12–16)	7233	14.11 (0.88)	14.21 (0.86)	14.01 (0.87)	0.22**
2. Domestic violence (0–3)	7206	0.02 (0.16)	0.05 (0.23)		
3. Psychological abuse (0–3)	7213	0.16 (0.34)	0.34/(0.43)		
4. Physical abuse (0–3)	7199	0.09 (0.29)	0.20/(0.40)		
5. Sexual abuse (0–3)	7196	0.04 (0.20)	0.08/(0.28)		
6. CSSI (0–3)	7169	0.60 (0.67)	0.84/(0.78)	0.39 (0.47)	0.70**
7. FA angry (0–50)	7241	21.75 (14.91)	22.34 (15.03)	21.09 (14.74)	0.08**
8. FA neutral (0–50)	7241	14.67 (13.16)	15.28 (13.25)	14.06 (12.99)	0.09**

FA Neutral = false alarm neutral, FA angry = false alarm angry

We observed significant differences between the two conditions in the emotional go/no-go task [i.e., FA angry vs. FA neutral; *t *(7179 = 35.28, *p* < 0.001)], showing that false alarm scores were higher when angry faces were presented as distractive stimuli compared to neutral distractors. This suggests an emotional interference effect when distractors (i.e., no-go trials) were presented as angry faces. Adolescents’ performance on the go/no-go task was comparable to reports from previous validation studies [49].

### Child maltreatment and somatic symptoms

In line with our first hypothesis, child maltreatment exposure was associated with somatic symptoms, accounting for the effect of age and gender. As presented in Table 2, when investigating relationships between types of child maltreatment exposure and somatic symptoms, only sexual abuse and psychological abuse were significantly associated with somatic symptoms.

**Table 2:** Regression tables on child maltreatment exposure and somatic symptoms

		*B*	SE	*β*	*P*
Model 1	Age	0.014	0.012	0.016	0.227
	Gender	− 0.371	0.020	− 0.251	0.000
	Domestic violence	0.094	0.056	0.025	0.092
	Psychological abuse	0.487	0.033	0.270	0.000
	Physical abuse	− 0.039	0.037	− 0.020	0.287
*R*^2^ = 0.159	Sexual abuse	0.199	0.041	0.070	0.000

*DV* = CSSI, FA neutral = false alarm neutral, FA angry = false alarm angry, CSSI = children’s somatic symptom inventory, *B* = regression coefficient, *β* = standardized regression coefficient, *R*^2^ = adjusted *R* square

### Affective inhibitory control and somatic symptoms

Relationships between affective inhibitory control and somatic symptoms in the total sample were investigated by conducting two separate regression analyses for each task condition (i.e., FA angry/FA neutral), adjusted for age and gender. In line with our second hypothesis, affective inhibitory control was significantly associated with somatic symptoms [*F* (3.7110) = 211.565, *p* < 0.001, *R*^2^ = 0.081] when angry stimuli were presented as no-go stimulus (FA angry: *β* = 0.086, *p* < 0.001), adjusted for age (*β* = 0.076, *p* < 0.001) and gender (*β* = − 0.276, *p* < 0.001). A significant association was also observed when angry stimuli was presented as go stimulus (FA neutral: *β* = 0.082, *p* < 0.001), adjusted for age (*β* = 0.078, *p* < 0.001) and gender (*β* = − 0.276, *p* < 0.001; *F* (3.7110) = 209.689, *p* < 0.001, *R*^2^ = 0.081). Significant relationships with age and gender indicate that higher somatic symptoms increases with age and that girls report higher somatic symptoms compared to boys. A significant relationship between affective inhibitory control and somatic symptoms was also observed when investigating the relationship in the maltreatment group only, for both FA neutral (*β* = 0.086, *p* < 0.001; *F* (3.3618) = 140.505, *p* < 0.001, *R*^2^ = 0.104) and FA angry (*β* = 0.061, *p* < 0.001; *F* (3.3618) = 135.281, *p* < 0.001, *R*^2^ = 0.100).

### Moderation effect of affective inhibitory control on types of child maltreatment and somatic symptoms

**Fig. 2:**
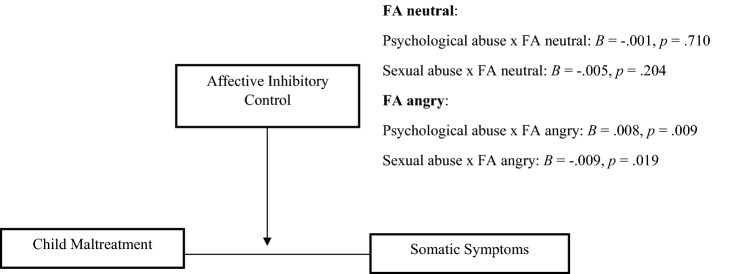
Moderation by affective inhibitory control on the relationship between psychological abuse and somatic symptoms, and sexual abuse and somatic symptoms

**Fig. 3:**
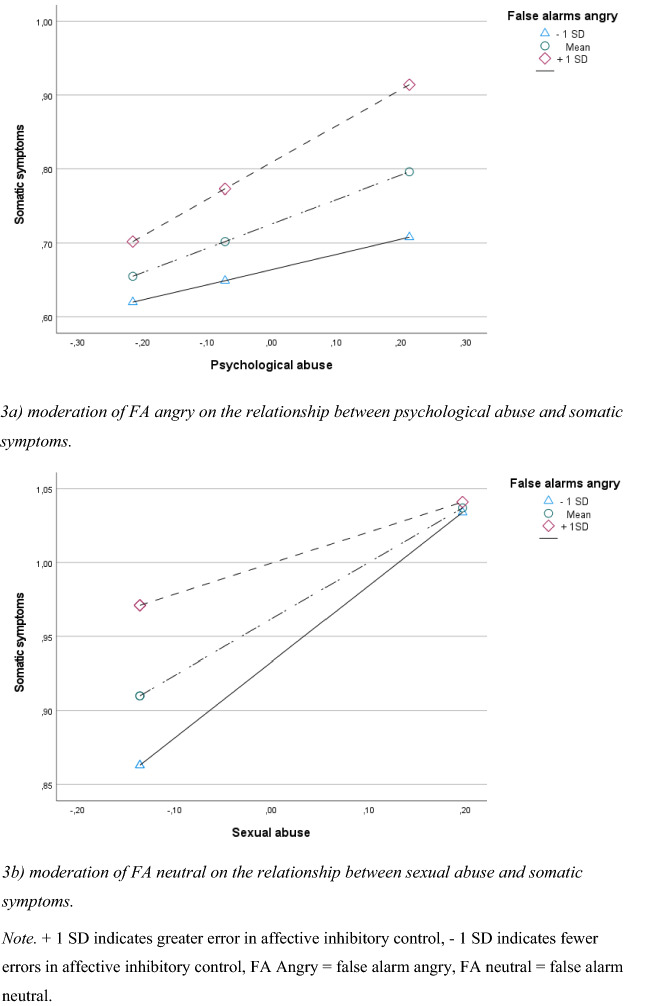
Simple slope investigation of moderation effects. Showing effects at low, moderate and high rates of affective inhibitory control problems on somatic symptoms. **a** Moderation of FA angry on the relationship between psychological abuse and somatic symptoms. **b** Moderation of FA neutral on the relationship between sexual abuse and somatic symptoms. + 1 SD indicates greater error in affective inhibitory control, − 1 SD indicates fewer errors in affective inhibitory control, FA angry = false alarm angry, FA neutral = false alarm neutral

Affective inhibitory control moderated the relationship between psychological abuse and somatic symptoms; however, only when angry facial expressions were presented as distractors (see Fig. 2). The relationship between psychological abuse and somatic symptoms was significant when affective inhibitory control was one SD below the mean (*B* = 0.220, *p* = 0.006), at the mean (*B* = 0.344, *p* < 0.001) and one SD above the mean (*B* = 0.469, *p* < 0.001). As shown in Fig. 3a, the strength of the relationship between psychological abuse and somatic symptoms increases along with problems with affective inhibitory control.

As shown in Fig. 2, affective inhibitory control moderated the relationship between sexual abuse and somatic symptoms as well. The relationship between sexual abuse and somatic symptoms was significant when affective inhibitory control was one SD below the mean (*B* < 0.489, *p* = 0.001), at the mean (*B* = 0.358, *p* < 0.001), and one SD above the mean (*B* = 0.229, *p* < 0.001). As indicated by the negative interaction term (see Fig. 2), the strength of the relationship between exposure to sexual abuse and somatic symptoms decreases as problems with affective inhibitory control increase (see Fig. 3). It is important to note that levels of somatic symptoms are considerably higher among those with greater problems with affective inhibitory control and low exposure to sexual abuse, compared to those with fewer affective inhibitory control problems, as shown in Fig. 3b.

## Discussion

This population-based study showed that exposure to child maltreatment increases the risk for somatic symptoms in adolescents. Significant relationships were observed for psychological abuse and sexual abuse, but not for physical abuse and witnessing domestic violence. Affective inhibitory control was associated with somatic symptoms regardless of a maltreatment history. Our findings suggest that adolescents report more somatic symptoms when also having greater problems with affective inhibitory control. With regard to the direct relationships between exposure to types of child maltreatment and somatic symptoms, affective inhibitory control moderated the relationship between exposure to psychological abuse and somatic symptoms, but only when angry emotional stimuli were presented as distractors. Moreover, the relationship between psychological abuse and somatic symptoms got stronger as affective inhibitory control problems increased. We also found a moderation effect for the relationship between child sexual abuse and somatic symptoms. However, contrary to our hypothesis, the interaction term was negative, suggesting that the strength of the positive relationship between exposure to sexual abuse and somatic symptoms decreased along with higher affective inhibitory control problems. This result suggests that individual differences in affective inhibitory control were more strongly related to differences in somatic symptoms at lower levels of sexual abuse exposure.

In the present study, adolescents with a history of exposure to child maltreatment displayed significantly higher levels of somatic complaints than their non-exposed peers, echoing findings from previous population-based data on adolescents with a history of maltreatment [1]. However, studies adjusting for co-occurrence of various types of abuse have so far been lacking with regard to the link to somatic symptoms in an adolescent sample. Both previous and present findings from population-based data suggest that different types of exposure may relate differently to somatic symptoms in adolescence [1]. Supporting this, and in line with other research on somatic symptoms after child maltreatment, both psychological and sexual abuse remained significantly related to somatic symptoms after adjusting for exposure to other types of abuse. In addition, the present finding shows a relatively stronger effect size for psychological abuse on somatic symptoms compared to other forms of abuse. This finding adds to the several studies highlighting psychological abuse as an equal or even stronger risk factor for psychopathology development compared to other types of abuse [e.g., 1, 30, 50].

Previous studies have shown that implicit emotion dysregulation, as indicated by problems with disengaging attention from emotional information, may increase the risk for somatic symptoms in clinical samples of adults [35, 36]. The present findings suggest that this might apply to adolescents as well. Moreover, unlike most other studies, we confirm this finding in a large-scale population-based study. Interestingly, we observed a significant relationship between affective inhibitory control and somatic symptoms when both neutral and angry emotional facial expressions were presented as distractors. This was somewhat unexpected, as our previous study on affective inhibitory control and internalizing symptoms (i.e., depression and anxiety) in maltreated adolescents only found inhibition of responses to emotional distractors to be significantly related to internalizing symptoms in female adolescents [30]. However, results are in line with other studies highlighting inhibitory control as an cognitive buffer against internalizing symptoms in high-risk populations [29]. In addition, findings are also in line with previous meta-analytic evidence suggesting that reduced inhibitory control performance in neutral conditions is related to various psychopathologies [51].

With regard to the moderating role of affective inhibitory control, results indicate that adolescents exposed to psychological abuse are at greater risk for experiencing somatic symptoms when they also experience greater difficulty inhibiting distracting emotional information. This aligns with previous studies suggesting that emotion dysregulation is a modulating mechanism between early life stress and psychopathology [30, 52, 53]. At the same time, the present findings further this research by highlighting the potential role of implicit ER in somatic symptoms. For the moderation effect observed for child sexual abuse, simple slopes analyses showed that the risk for somatic symptoms was greater for those with poorer affective inhibitory control. The influence of affective inhibitory control seemed, however, to be reduced as the degree of exposure increased. Although this finding requires further investigation, this may reflect an important etiological characteristic of somatic symptom development in adolescents exposed to child sexual abuse. This finding may suggest that individual differences in implicit regulatory processes influence symptom development at the low levels of exposure, but may be reduced as the frequency and severity of sexual abuse increases.

Effect sizes for all identified relationships were small, with questionable clinical significance. Nevertheless, revealing this relationship in a low-risk population-based sample may indicate that interventions targeting affective inhibitory control could be beneficial in clinical settings and should be explored further. The importance of affective inhibitory control for symptom development at even low levels of exposure and symptomatology may indicate a potential benefit of early intervention and prevention of symptom development after certain types of child maltreatment. Preliminary evidence suggests that such interventions may facilitate more adaptive ER and reduce post-trauma symptoms [19]. Moreover, as both somatic complaints and exposure to child maltreatment are relatively common in the general population and impact wellbeing and functioning throughout life [4, 6, 14], a greater understanding of the mechanisms involved is warranted from a public health perspective.

### Limitations and future directions

The cross-sectional design of the present study has some methodological shortcomings. No causal interpretation of the direction of the identified relationships can be made, and prospective data are needed to address causality. As this is an epidemiological study with a non-clinical sample, generally low levels of somatic symptoms and exposure to child maltreatment were reported. This may also explain the small but significant effect sizes. We did not distinguish between different somatic symptoms, and future studies should investigate whether the relationship with affective inhibitory control varies with specific symptoms (e.g., chronic pain vs. fatigue). In addition, affective inhibitory control is just one of several important components in adolescent ER [18]. Other components of ER, such as mentalization [54], emotion recognition [55] and cognitive ER strategies [26, 55], are also related to the risk for somatic symptoms. Child maltreatment also represents a complex and heterogeneous phenomenon that is accompanied by several methodological challenges [56]. Future studies should test how other characteristics of child maltreatment exposure (e.g., timing, duration) relate to both implicit ER and somatic symptoms.

## Conclusions

Collectively, findings from this study suggest that implicit emotion dysregulation is a modulating mechanism between exposure to child maltreatment and somatic symptoms. The findings expand on the growing body of research suggesting that explicit and implicit emotion dysregulation is related to elevated levels of persistent somatic symptoms [15]. Among adolescents exposed to psychological abuse, the risk for somatic symptoms increased along with greater affective inhibitory control problems. For exposure to child sexual abuse, findings indicated that the protective role of inhibitory control was only present at low levels of exposure and somatic symptoms. Perhaps indicating that for this type of abuse, affective inhibitory control is less of a protective asset as exposure severity increase. Revealing these associations in a population-based sample highlights potential etiological differences for the impact of sexual abuse and psychological abuse on somatic symptoms. Results also suggest that treatment targeting affective inhibitory control may be beneficial and should be explored further in clinical settings.

## Supplementary Information

Below is the link to the electronic supplementary material.

Supplementary file1 (DOCX 30 KB)
