# Correction to: The regulatory role of affective inhibitory control in somatic symptoms among adolescents exposed to child maltreatment: a population-based study

**DOI:** 10.1007/s00787-022-02008-4

**Published:** 2022-05-19

**Authors:** Sjur Skjørshammer Sætren, Else-Marie Augusti, Mia Cathrine Myhre, Gertrud Sofie Hafstad

**Affiliations:** 1 Department for Child and Adolescent Research, Norwegian Centre for Violence and Traumatic Stress Studies, Oslo, Norway; 2 TIPS Centre for Clinical Research in Psychosis, Stavanger University Hospital, Jan Johnsens gate 12, 4011 Stavanger, Norway

## Correction to: European Child & Adolescent Psychiatry 10.1007/s00787-022-01988-7

The article “The regulatory role of affective inhibitory control in somatic symptoms among adolescents exposed to child maltreatment: a population-based study”, written by Sjur Skjørshammer Sætren, Else-Marie Augusti, Mia Cathrine Myhre, Gertrud Sofe Hafstad was originally published electronically on the publisher’s internet portal (currently SpringerLink) on April 20, 2022 without open access. This article is licensed under a Creative Commons Attribution 4.0 International License, which permits use, sharing, adaptation, distribution and reproduction in any medium or format, as long as you give appropriate credit to the original author(s) and the source, provide a link to the Creative Commons licence, and indicate if changes were made. The images or other third party material in this article are included in the article’s Creative Commons licence, unless indicated otherwise in a credit line to the material. If material is not included in the article’s Creative Commons licence and your intended use is not permitted by statutory regulation or exceeds the permitted use, you will need to obtain permission directly from the copyright holder. To view a copy of this licence, visit http://creativecommons.org/licenses/by/4.0/.

The original article has been corrected.

