# Supplementary material for: The regulatory role of affective inhibitory control in somatic symptoms among adolescents exposed to child maltreatment: a population-based study
Source: Eur Child Adolesc Psychiatry. 2022 Apr 20;32(9):1701–10. doi: 10.1007/s00787-022-01988-7 (PMC10460330; doi:10.1007/s00787-022-01988-7)
Supplement: Supplementary file 1 — Supplementary file1 (DOCX 30 KB) [file 787_2022_1988_MOESM1_ESM.docx]

**Table 1.** *Pearson correlations among all variables*

|  |  | 1 | 2 | 3 | 4 | 5 | 6 | 7 | 8 |
| --- | --- | --- | --- | --- | --- | --- | --- | --- | --- |
| 1. Age (12 – 16) |  | – |  |  |  |  |  |  |  |
| 1. Domestic violence   (0-3) |  | .027^**^ | – |  |  |  |  |  |  |
| 1. Psychological abuse (0-3) |  | .076^**^ | .378^**^ | – |  |  |  |  |  |
| 1. Physical abuse (0-3) |  | .042^**^ | .414^**^ | .673^**^ | – |  |  |  |  |
| 1. Sexual abuse (0-3) |  | .035^**^ | .213^**^ | .335^**^ | .386^**^ | – |  |  |  |
| 1. CSSI (0-3) |  | .068^**^ | .168^**^ | .368^**^ | .250^**^ | .179^**^ | – |  |  |
| 1. FA angry (0-50) |  | -.076^**^ | .017 | .047^**^ | .037^**^ | .046^**^ | .043^**^ | – |  |
| 1. FA neutral (0-50) |  | -.105^**^ | .043^**^ | .064^**^ | .064^**^ | .060^**^ | .039^**^ | .266^**^ | – |

*Note.* All pairwise, Pearson correlations ***p <.*01. * *p* <.05, Domestic violence = witnessing domestic violence, CSSI = children’s somatic symptom inventory, FA Neutral = false alarm neutral, FA angry = false alarm angry.

**Table 2.** *T-test comparing female and male adolescents on all variables*

|  | Girls | | Boys | |  |  |  |
| --- | --- | --- | --- | --- | --- | --- | --- |
|  | M | SD | M | SD | t | p | Cohen’s *d* |
| Domestic violence | .02 | .15 | .01 | .14 | 2.64 | .008 | .06 |
| Psychological abuse | .18 | .36 | .12 | .30 | 6.40 | .000 | .06 |
| Physical abuse | .09 | .29 | .08 | .25 | 1.72 | .085 | .04 |
| Sexual abuse | .04 | .20 | .02 | .14 | 3.72 | .000 | .08 |
| CSSI | .77 | .74 | .42 | .51 | 22.72 | .000 | .54 |
| FA neutral | 13.12 | 12.17 | 16.44 | 13.97 | -10.75 | .000 | .25 |
| FA angry | 19.93 | 14.49 | 23.74 | 15.25 | -10.88 | .000 | .25 |

*Note. M*, mean. *SD,* standard deviation. Domestic violence = witnessing domestic violence, CSSI = children’s somatic symptom inventory, FA Neutral = false alarm neutral, FA angry = false alarm angry.
